# Current News

**Published:** 1899-04

**Authors:** 


### Current News.

### NEW ENGLAND ASSOCIATION OF DENTAL EXAMINERS.

The Third Annual Meeting and dinner of the New England Association was held at the Algonquin Club, in Boston, on the evening of February 16, 1899. Dr. John F. Dowsley, President of the Massachusetts Board, presiding. There were present as guests of the Association Dr. L. D. Shepard, of Boston, and Dr. C. A. Brackett, of Newport. Regrets were read from Dr. C. A.

Meeker, Secretary of the National Association; Dr. Civilion Fones, of Connecticut, and Dr. J. Searle Hurlbut, of Massachusetts, who had hoped to be present.

Great interest was taken in the object for which this Association has been formed,—namely, to raise the standard of examinations in New England to one of uniform requirements,—and as a result two of the New England States have had their dental laws so amended as to bring them in line with their sister States. It is the ardent hope of the Association that in course of time a law so nearly perfect will be enacted as to render feasible an interchange of certificates in New England.

An election of officers for the ensuing year resulted in the choice of Dr. D. F. Keefe, of Providence, R. I., President; G. F. C. Cheeney, of St. Johnsbury, Vt., Vice-President; Geo. L. Parmele, of Hartford, Conn., Recorder. The following ex-members of State boards were elected honorary members: Dr. C. A. Brackett, of Rhode Island; Dr. J. Searle Hurlbut, of Massachusetts; Dr. Civilion Fones, of Connecticut; Dr. L. D. Shepard, of Massachusetts; Dr. R. B. Miller, formerly of Maine, and Dr. James Lewis, of Vermont.

GEO. L. PARMELE,
*Recorder.*

---

### DENTAL SOCIETY, STATE OF NEW YORK.

THE Thirty-first Annual Meeting of the above Society will be held in Albany, May 10 and 11, 1899, and the following programme will be presented:

President's Address.
Report of Correspondent, R. Ottolengui, M.D.S.
Report of Committee on Practice, L. C. Le Roy, D.D.S.
Essay, L. D. Shepard, M.D., D.D.S., Boston, Mass.
Essay, R. M. Sanger, D.D.S., East Orange, N. J.
Essay, William Hailes, Jr., M.D., Albany.
Essay, A. Retter, D.D.S., Utica.

A cordial invitation is extended to all reputable dentists to attend the meeting.

F. LE GRAND AMES, *President.*
CHARLES S. BUTLER, *Secretary.*

